# A162 CHRONIC OPIOID THERAPY IS ASSOCIATED WITH INCREASED HEALTH SERVICES UTILIZATION AND DIRECT HEALTHCARE COSTS: A POPULATION-BASED STUDY

**DOI:** 10.1093/jcag/gwab049.161

**Published:** 2022-02-21

**Authors:** E Kuenzig, J Mason, C N Bernstein, T Gomes, D Juurlink, G G Kaplan, J Peña-Sánchez, L E Targownik, S Vigod, J Begum, Z Nugent, E I Benchimol

**Affiliations:** 1 Gastroenterology, Hepatology and Nutrition, The Hospital for Sick Children, Department of Pediatrics, University of Toronto, Toronto, ON, Canada; 2 Centre for Addiction and Mental Health, Toronto, ON, Canada; 3 Internal Medicine, University of Manitoba, Winnipeg, MB, Canada; 4 Uiversity of Manitoba, Winnipeg, MB, Canada; 5 University of Toronto, Toronto, ON, Canada; 6 Medicine and Community Health Sciences, University of Calgary, Calgary, AB, Canada; 7 Department of Community Health and Epidemiology, University of Saskatchewan, Saskatoon, SK, Canada; 8 Women’s College Hospital, Toronto, ON, Canada; 9 ICES, Toronto, ON, Canada

## Abstract

**Background:**

Opioid use is more common among people living with Inflammatory bowel disease (IBD).

**Aims:**

Investigate the associations between receiving chronic opioid therapy and health services utilization and direct healthcare costs among IBD patients receiving chronic opioid therapy.

**Methods:**

We identified prevalent cases of IBD in Ontario (7/2012–3/2017) from population-based health administrative data using previously validated algorithms. Patients with ongoing opioid use for >90 days (chronic opioid recipients) were matched based on age, sex, IBD type, and disease duration with up to 5 IBD patients with no period of chronic opioid use. For the year after becoming a chronic opioid recipient (i.e., from the 91^st^ day of chronic opioid therapy), we determined: 1) the mean (SD) number of IBD-specific, IBD-related, and all-cause outpatient visits, emergency department (ED) visits, and hospitalizations; and 2) total, hospitalization, ED, and outpatient healthcare costs. IBD-specific visits had a diagnostic code for Crohn’s disease or ulcerative colitis; IBD-related visits additionally included diagnostic codes for signs, symptoms, and extra-intestinal manifestations of IBD. Patients were censored at the time of a new cancer diagnosis or upon initiation of palliative care. We used Poisson models to compare the number of healthcare encounters in chronic opioid recipients and patients with no period of chronic opioid therapy then used generalized linear models with a gamma distribution and log-link to compare direct healthcare costs in the two groups. Regression models accounted for matching and were adjusted for income, rural/urban household, and comorbidities (resource intensity using the John Hopkins ACG Index).

**Results:**

We identified 9913 IBD patients with at least one period of chronic opioid therapy matched to 44,274 without chronic opioid therapy (mean 43 y at chronic opioid use, 43% male, 58% Crohn’s). Patients receiving chronic opioid therapy had significantly more health care encounters (Figure A). Annual per capita total health care cost among chronic opioid recipients was $13,452 (SD 33,777) compared to $5140 (SD 28,999) among patients with no chronic opioid therapy (Figure B). After adjustment, healthcare costs were approximately double in chronic opioid recipients and was consistent for all cost types (overall, hospitalization, ED, and outpatient).

**Conclusions:**

IBD patients who were chronic opioid users had significantly more health services utilization and direct healthcare costs compared to patients without periods of chronic opioid use. These associations persisted after adjusting for the resource intensity of any co-occurring conditions.

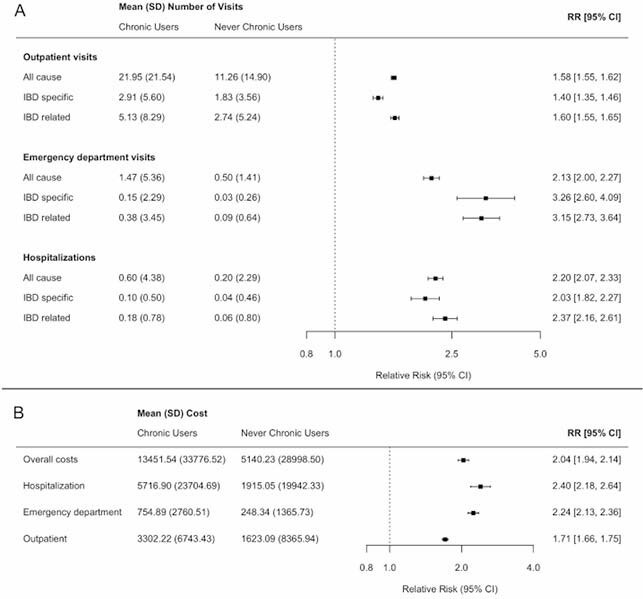

Health services utilization (A) and direct healthcare costs (B) among people with inflammatory bowel disease (IBD) who are chronic opioid users compared to those without a period of chronic opioid use.

**Funding Agencies:**

American College of Gastroenterology

